# Digital fetal scalp stimulation (dFSS) versus fetal blood sampling (FBS) to assess fetal wellbeing in labour—a multi-centre randomised controlled trial: Fetal Intrapartum Randomised Scalp Stimulation Trial (FIRSST NCT05306756)

**DOI:** 10.1186/s13063-022-06794-9

**Published:** 2022-10-04

**Authors:** D. J. Murphy, Y. Shahabuddin, S. Yambasu, K. O’Donoghue, D. Devane, A. Cotter, G. Gaffney, L. A. Burke, E. J. Molloy, F. Boland

**Affiliations:** 1 Academic Department of Obstetrics and Gynaecology, Coombe Women & Infants University Hospital & Trinity College, University of Dublin, Dublin, Ireland; 2 Pregnancy Loss Research Group, Department of Obstetrics & Gynecology, University College Cork, Cork, Ireland; 3 INFANT Research Centre, University College Cork, Cork, Ireland; 4 University of Galway, School of Nursing and Midwifery, HRB-Trials Methodology Research Network, Evidence Synthesis Ireland and Cochrane Ireland, Galway, Ireland; 5 Department of Obstetrics and Gynecology, University of Limerick, Limerick, Ireland; 6 Department of Obstetrics and Gynaecology, University of Galway, Galway, Ireland; 7 Department of Economics, Cork University Business School, University College Cork, Cork, Ireland; 8 Department of Paediatrics, Trinity College Dublin, Dublin, Ireland; 9 Data Science Centre and the Department of General Practice, RCSI, Dublin, Ireland

**Keywords:** Digital fetal scalp stimulation (dFSS), Fetal blood sampling (FBS), Labour, Cardiotocography, Caesarean section, Randomised controlled trial

## Abstract

**Background:**

Cardiotocography (CTG) is a screening test used to detect fetal hypoxia in labour. It has a high false positive rate resulting in many potentially unnecessary caesarean sections. Fetal blood sampling (FBS) is a second-line test of the acid-base status of the fetus. It is used to provide either reassurance that it is safe for labour to continue or objective evidence of compromise so that delivery can be expedited. Digital fetal scalp stimulation (dFSS) to elicit a fetal heart rate acceleration is an alternative less invasive second-line test of fetal wellbeing. This study aims to provide robust evidence on the role of these two second-line tests in assessing fetal wellbeing and potentially preventing operative delivery.

**Methods:**

A multi-centre parallel group randomised controlled trial (RCT) is planned in four maternity centres in Ireland. The study aims to recruit 2500 nulliparous women with a term (≥37+0 weeks) singleton pregnancy who require a second-line test of fetal wellbeing in labour due to an abnormal CTG. Women will be allocated randomly to dFSS or FBS on a 1:1 ratio. The primary outcome is caesarean section. With 1250 women in each arm, the study will have 90% power to detect a difference of 5–6%, at a two-sided alpha significance level of 5%, assuming a caesarean section rate of at least 20% in the dFSS group.

**Discussion:**

If the proposed study shows evidence that dFSS is a safe, reliable and effective alternative to FBS, this would have ground-breaking implications for labour management worldwide. It could potentially lead to a reduction in invasive procedures and emergency caesarean sections.

**Trial registration:**

ClinicalTrials.gov NCT05306756. Registered on 31 March 2022. The trial commenced enrolment on 10 May 2022. Ethical committee approval has been granted by the Research Ethics Committee (REC) of each hospital: Dublin/CWIUH REC: 12.06.2019; Cork/UCC REC: 29.11.2019; Galway/NUIG REC: 06.09.2019; Limerick/UL REC: 30.09.2019.

## Administrative information

Note: the numbers in curly brackets in this protocol refer to SPIRIT checklist item numbers. The order of the items has been modified to group similar items (see http://www.equator-network.org/reporting-guidelines/spirit-2013-statement-defining-standard-protocol-items-for-clinical-trials/).

**Table:** 

Title {1}	Digital Fetal Scalp Stimulation (dFSS) versus Fetal Blood Sampling (FBS) to assess fetal wellbeing in labour – a multi-centre randomised controlled trial. Acronym: FIRSST - Fetal Intrapartum Randomised Scalp Stimulation Trial
Trial registration {2a and 2b}.	ClinicalTrials.gov Identifier: NCT05306756. Registered Prospectively on 31 March 2022. Trial commenced enrolment 10 May 2022.
Protocol version {3}	Version 9.0; Date 03.08.22
Funding {4}	Health Research Board of Ireland funded through the Definitive Interventions and Feasibility Awards (DIFA) 2019.
Author details {5a}	Deirdre J Murphy, Professor of Obstetrics, Academic Department of Obstetrics and Gynaecology, Coombe Women & Infants University Hospital & Trinity College, University of Dublin, Ireland.
	Yulia Shahabuddin, Trial Co-ordinator, Academic Department of Obstetrics and Gynaecology, Coombe Women & Infants University Hospital & Trinity College, University of Dublin, Ireland.
	Sahr Yambasu, Research Fellow, Academic Department of Obstetrics and Gynaecology, Coombe Women & Infants University Hospital & Trinity College, University of Dublin, Ireland.
	Keelin O’Donoghue, Professor of Obstetrics, Pregnancy Loss Research Group, Department of Obstetrics & Gynecology, University College Cork, & INFANT Research Centre, University College Cork, Cork, Ireland.
	Declan Devane, Professor of Health Research Methodology, University of Galway, School of Nursing and Midwifery, HRB- Trials Methodology Research Network, Evidence Synthesis Ireland and Cochrane Ireland.
	Amanda Cotter, Professor of Obstetrics, Department of Obstetrics and Gynecology, University of Limerick, Limerick, Ireland.
	Geraldine Gaffney, Senior Lecturer & Consultant Obstetrician & Gynaecologist, Department of Obstetrics and Gynaecology, University of Galway, Galway, Ireland.
	Lee-Ann Burke, Lecturer in Health Economics, Department of Economics, Cork University Business School. University College Cork, Cork, Ireland.
	Eleanor Molloy, Professor of Paediatrics and Child Health, Department of Paediatrics, Trinity College Dublin, Dublin, Ireland.
	Fiona Boland, Senior Lecturer in Biostatistics, Data Science Centre and the Department of General Practice, RCSI, Dublin, Ireland.
Name and contact information for the trial sponsor {5b}	Sponsor: Trinity College, University of Dublin, Dublin 2, Ireland. DIFA 2018-019.
Role of sponsor {5c}	The study sponsor and funders have no role in the design of the study and collection, analysis and interpretation of data and in writing the manuscript.

## Background and rationale {6a}

### Background and rationale

The aim of fetal heart rate monitoring in labour is to detect hypoxia that can lead to fetal acidosis, hypoxic ischaemic encephalopathy, irreversible brain injury and peripartum death [1]. Continuous electronic fetal monitoring by cardiotocography (CTG) has a high sensitivity for intrapartum fetal hypoxia and acidosis but also a high false positive rate [1, 2]. The false positive rate is reported to be of the order of 60%, resulting in many potentially unnecessary caesarean sections (CS) and assisted vaginal births (AVB) [1, 3]. Second-line tests are used to provide either reassurance that it is safe for labour to continue or objective evidence of compromise so that delivery can be expedited [1, 4–6]. In theory, if fetal wellbeing is confirmed, this should reduce the incidence of operative delivery. Fetal blood sampling (FBS) is used to test the acid-base status of the fetus from a scalp capillary blood sample. Digital fetal scalp stimulation (dFSS) is an alternative approach where the fetal scalp is rubbed at the vaginal examination to elicit a fetal heart rate (FHR) acceleration. An FHR acceleration provides reassurance about fetal wellbeing [5, 7]. This trial aims to compare dFSS and FBS in women with term singleton pregnancies and an abnormal intrapartum CTG, where additional information on fetal wellbeing is required.

#### Evidence in relation to FBS

Fetal blood sampling is an invasive procedure, where a few drops of capillary blood are collected in heparinised tubes following a small fetal scalp puncture with a blade [3]. When fetal hypoxia occurs, anaerobic metabolism results in a state of metabolic acidosis and fetal blood pH falls due to the build-up of hydrogen ions. An abnormal pH is classified as less than 7.20 and this threshold has been shown to have a higher specificity than a pathological CTG in predicting a low Apgar score at 1 min [1]. This threshold is used to indicate the need for urgent delivery. A Cochrane systematic review concluded that there is no evidence that FBS as an adjunct to CTG monitoring reduces the incidence of emergency caesarean delivery or influences the reduction in neonatal seizures associated with continuous CTG monitoring [1]. Despite this, the Intrapartum Care Guidelines of the National Institute for Health and Care Excellence (NICE) recommend that a fetal blood sample should be performed in the presence of a pathological CTG before expediting delivery [4].

Recent studies have questioned the validity and reliability of FBS and highlighted the logistic challenges of achieving a reliable result in a timely manner [8–11]. A study reported a median time of 18 min to achieve a result. In 9% of cases, the time taken exceeded 30 min, with an adequate sample for analysis collected in only 79% of cases [10]. A further study reported that the median time interval was 37 min from the decision to perform the FBS to delivery following an abnormal result and that slower time intervals were associated with maternal body mass index (BMI) greater than 25, cervical dilatation less than 5 cm and less experienced operators [8]. A prospective cohort study from our group evaluated 100 FBS procedures with contemporaneous paired samples [11]. There was a significant difference between the mean pH of the two samples and in 43 pairs the difference was greater than the laboratory acceptable maximum analytical difference. There was discordance in the recommended clinical decision between 16 paired samples and in 11 of these cases delivery was by emergency caesarean section.

#### Evidence in relation to dFSS

Fetal scalp stimulation by digital rubbing (dFSS) has been suggested as an alternative less invasive supplementary test of fetal wellbeing in labour [5, 7]. An acceleration of the fetal heart rate following fetal scalp stimulation indicates that the likelihood of a low scalp pH is 2% [7]. Digital FSS has a number of potential advantages. It is non-invasive to the fetus and can be incorporated into a standard vaginal examination performed by a midwife or obstetrician. The result will be available within 5 to 10 min and can be interpreted by staff with the skillset to interpret a CTG. It can be used in all labouring women including those with a contraindication to FBS such as suspected fetal bleeding disorders or where the cervix is less than 3cm dilated, and it has no additional cost. More recently, it has been recommended in Irish and UK guidelines, with the acknowledgement that evidence is lacking [4, 12].

#### Evidence comparing FBS and dFSS

We conducted a prospective cohort study of 299 consecutive second-line tests comparing FBS and FSS [13]. There was a strong correlation between the FBS and FSS results suggesting that in most cases the FSS test would be as reliable as FBS. In the cases where the FBS result was abnormal (pH<7.20) and the FSS was reassuring (potential false negatives), the cord blood results and Apgar scores were within the normal range. While these data are insufficient to change practice, they support the need for robust research to address the question whether dFSS could replace FBS as a test of fetal wellbeing in labour.

We completed a feasibility study at the Coombe Women & Infants University Hospital from Aug 2017 to Jan 2018 [14]. There was a high rate of participation demonstrating a willingness among women to contribute to the study. We recruited 66 women achieving full protocol adherence in 50 cases. It was clear from a feasibility perspective that we should limit the study to nulliparous women where eligibility for randomisation was greater due to longer labours and more frequent CTG abnormalities requiring second-line testing.

We followed the feasibility study with a pilot randomised controlled trial completed over a 4-month period. We recruited and randomised a total of 50 patients which informed our estimates for recruitment over the lifetime of the definitive trial. The caesarean section rate was 20% in the dFSS group and more than twice that in the FBS group. The feasibility and pilot trial allowed us to refine our study procedures and sample size calculations for a definitive trial. Health professionals involved in patient care were supportive of the trial and adapted quickly to the trial interventions.

#### Cochrane systematic review

A Cochrane systematic review titled “Fetal scalp stimulation for assessing fetal wellbeing during labour” evaluated fetal scalp stimulation compared to other tests of fetal wellbeing and to CTG alone [15]. The Cochrane search identified only two eligible studies: the first our pilot study (50 participants) and the second a trial from India that compared CTG and manual fetal stimulation with CTG alone (327 participants). The review concluded that there is only very low-certainty evidence currently in relation to fetal scalp stimulation as an approach for assessing fetal wellbeing in labour, and further well-designed RCTs are required.

## Objectives {7}

### Objectives

The aim of this study is to compare digital fetal scalp stimulation (dFSS) with fetal blood sampling (FBS) as second-line tests of fetal wellbeing in labour. The hypothesis is that digital fetal scalp stimulation (dFSS) performs better than fetal blood sampling (FBS) in terms of reducing the rate of emergency caesarean section in labour, without adversely affecting perinatal outcomes.

#### Primary objective

- To perform a robust multi-centre randomised controlled trial to compare the effect of dFSS versus FBS on the CS rate for nulliparous women at term (37 weeks or more) with CTG abnormalities that require a second-line test of fetal wellbeing

#### Secondary objectives

- To assess the effect on assisted vaginal birth (AVB)
- To assess the effect on maternal and perinatal morbidity and mortality outcomes
- To assess the effect on procedural outcomes
- To assess the maternal acceptability of the procedures
- To explore any variation in effect relating to the presence of meconium or labour induction
- To perform a health-economics analysis

## Trial design {8}

### Trial design

Multi-centre parallel group randomised controlled trial with women randomised to fetal blood sampling (FBS) or digital fetal scalp stimulation (dFSS) in a ratio of 1:1, testing whether dFSS is superior to FBS.

## Methods: participants, interventions and outcomes

### Study setting {9}

The study will be conducted in four university-affiliated maternity units in Ireland encompassing almost 22,000 births per annum. Approximately 10% of women in labour require second-line testing by FBS.

**Table:** 

Coombe Women & Infants University Hospital (CWIUH)	7500–8000 births
Cork University Maternity Hospital	7000–7500 births
University Maternity Hospital Limerick	4500 births
University Maternity Hospital Galway	2500 births

### Eligibility criteria {10}

#### Inclusion

The study will be limited to nulliparous women with a singleton pregnancy, cephalic presentation, gestational age 37+0 weeks or greater and an abnormal CTG that requires further assessment by fetal blood sampling or digital fetal scalp stimulation. The allocated second-line test will be performed and interpreted by an obstetrician.

#### Exclusion

Multiparous women or nulliparous women with a contraindication to FBS, or who have a limited understanding of English or are under 18 years of age. Eligibility will also be at the discretion of the responsible obstetrician in cases where there is urgency due to suspected fetal compromise (e.g. prolonged fetal bradycardia).

### Who will take informed consent? {26a}

#### Informed consent

An information leaflet about the study will be provided to all nulliparous women at a routine antenatal clinic visit. Further information will be provided at birth education classes and posters will be placed in relevant clinical settings to raise awareness about the study.

Recruitment of women to the study will follow a three-stage process.

1. All potentially eligible women will be given written information about the study prior to consent. The patient information leaflet (PIL) will explain the trial purpose and design, making it clear that women are only eligible for the study if they require a continuous CTG in labour and subsequently if a second-line test is required due to CTG abnormalities. The leaflet will contain contact details to allow women to discuss the study further if they wish.
2. Once a woman has presented for induction of labour or in early labour, a trained doctor or midwife will seek written informed consent if the following criteria are satisfied:
  i) The midwife looking after the woman assesses her to be capable of providing informed consent.
  ii) The woman has adequate pain control.
  iii) The woman has not used systemic opiates in the last 4 h.
3. Once written consent has been given, the mother will not be consulted again unless she requires a second-line test in labour due to an abnormal CTG. After confirmation that all criteria are met and that the woman is happy to continue in the study, the doctor or midwife will obtain the randomised allocation.

#### Allocation to trial groups

Allocation of eligible women who consent to participate in the trial will be concealed using a fully automated password-protected computer-based system provided by the HRB Centre for Primary Care Research, Royal College of Surgeons in Ireland.

It is not possible to blind the participating woman or treating staff. The outcome assessment will be blinded for the primary and secondary clinical outcomes. The researcher will complete the case report form (CRF) for the primary and secondary maternal and perinatal outcomes from the computerised records without knowledge of the allocation. Only when these parts of the dataset are complete will they review the detailed intrapartum records to record the procedural variables relating to second-line testing for CTG abnormalities.

### Additional consent provisions for collection and use of participant data and biological specimens {26b}

Women will be asked to give permission for data to be stored for possible future research related to the current study (fetal wellbeing in labour) without further consent being required but only if the research is approved by a Research Ethics Committee. This trial does not involve collecting biological specimens for storage.

## Interventions

### Explanation for the choice of comparators {6b}

All women will have continuous electronic fetal heart rate monitoring by CTG. The intervention of interest is digital fetal scalp stimulation (dFSS) which will be compared to fetal blood sampling (FBS). Other methods of fetal stimulation have been described including pinching the scalp, application of an Allis forceps to the scalp and vibroacoustic stimulation [7, 16]. Digital FSS has been chosen as it is non-invasive to the fetus, easy to perform and minimally uncomfortable to the patient when performed as part of a vaginal examination. The comparator is fetal blood sampling which has been part of routine practice in most Irish maternity centres for many years. Until recently, FBS was considered the gold standard second-line test of fetal wellbeing in labour, as reflected in clinical guidelines [8].

### Intervention description {11a}

#### Intervention

##### FBS arm

Women allocated to FBS will be managed according to NICE/RCOG guidelines and the local hospital protocol [4]. The women will be assessed by abdominal and digital vaginal examination prior to FBS. Once the decision to perform a fetal blood sample has been made, fetal capillary blood samples will be collected in heparinised tubes and analysed in the delivery suite using the locally available gas analyser. The result of the first technically reliable sample will be interpreted and acted upon according to the protocol, or if multiple samples are tested the lowest technically reliable result will be used, taking account of the clinical circumstances and the stage of labour:

**Table:** 

pH ≥ 7.25	normal	continue and if indicated repeat in 60 min
pH 7.21– 7.24	borderline	repeat in 30 min
pH ≤ 7.20	abnormal	deliver

##### dFSS arm

Women allocated to dFSS will be managed in the same way except dFSS will be performed instead of FBS. An abdominal and vaginal assessment will be performed as usual. The examiner will stimulate the fetal scalp digitally with the index and middle finger for 30–60 s [5]. The woman will be optimally positioned avoiding aorto-caval compression (tilted towards the left lateral). The CTG will be observed over a 5- to 10-min interval after the dFSS, and if a fetal heart rate acceleration (>15 bpm for 15 s) is observed, the dFSS test will be considered normal and should be interpreted in the same way as a normal pH result following FBS. If there is no acceleration and an episode of normal fetal heart rate variability (5–25 bpm), this is classified as borderline and dFSS should be repeated in 30 min as with a borderline pH result. If there is no fetal heart rate acceleration and no episode of normal variability with ongoing abnormal features, the dFSS should be interpreted as abnormal in the same way as an abnormal FBS result and warrants expedited delivery in keeping with the clinical circumstances (or consideration of FBS).

**Table:** 

Acceleration ≥15bpm for 15 s	normal	if indicated repeat in 60 min
Uncertain acceleration/normal variability	borderline	repeat in 30 min
No acceleration/ongoing abnormal features	abnormal	deliver/or consider FBS

### Criteria for discontinuing or modifying allocated interventions {11b}

Once the patient has received an allocation of either FBS or dFSS, this should be the preferred second-line test if ongoing assessments are required. In all cases of either FBS or dFSS, the results need to be interpreted as part of the full clinical picture. If the result seems out of keeping with the full clinical picture, or an alternative course of action is indicated, this needs to be discussed with the lead obstetrician. If the dFSS is inconclusive, uninterpretable or abnormal, the obstetrician may proceed to FBS if they wish.

### Strategies to improve adherence to interventions {11c}

All labour ward staff in Irish maternity units are required to complete mandatory training on CTG interpretation. A standard operating procedure will be provided at each site to ensure consistency in the approach to performing second-line tests. Staff education will be ongoing throughout the study to promote adherence to the intervention protocols and adherence will be monitored on a monthly basis for at least the first year of the study. Deviation from the allocated second-line test will be recorded in the case report form (CRF).

### Relevant concomitant care permitted or prohibited during the trial {11d}

Decisions relating to patient safety take precedence over all trial procedures.

### Provisions for post-trial care {30}

There is no anticipated harm and compensation for trial participation.

### Outcomes {12}

Primary outcome:

1. Caesarean section (CS)
  All caesarean sections will be in labour in the context of an abnormal CTG [time frame: at birth]

Secondary outcomes:

2. Caesarean section, primary indication fetal concerns — abnormal CTG, or meconium, or low pH on FBS [time frame: at birth]
3. Caesarean section, primary indication poor progress in first or second stage of labour [time frame: at birth]
4. Caesarean section, failed attempt at assisted vaginal birth in the second stage of labour [time frame: at birth]
5. Assisted vaginal birth (AVB) (all cases) — vacuum or forceps or sequential (vacuum and forceps) [time frame: at birth]
6. Assisted vaginal birth, primary indication fetal concerns — abnormal CTG, or meconium, or low pH on FBS [time frame: at birth]
7. Assisted vaginal birth, primary indication of poor progress in the second stage of labour [time frame: at birth]
8. Spontaneous vaginal birth (SVB) [time frame: at birth]
9. Decision delivery interval (DDI) for emergency CS >30 min — DDI prolonged [time frame: interval between decision time and time of birth]
10. Decision delivery interval (DDI) for AVB >15 min — DDI prolonged [time frame: interval between decision time and time of birth]
11. Perinatal death — intrapartum or early neonatal death [time frame: up to 7 days of age]
12. Late perinatal death — after 7 days up to 28 days of age [time frame: 8–28 days of life]
13. Apgar score at 5 min <7 — low Apgar score at 5 min [time frame: age 5 min]
14. pH umbilical artery <7.00 or base excess artery <−12.0 — arterial cord blood acidosis [time frame: immediately after birth]
15. Admission to the neonatal unit (NNU) — admission all causes [time frame: after birth]
16. Neonatal encephalopathy (Sarnat definition grades I–III) [time frame: after birth]
17. Therapeutic hypothermia treatment for encephalopathy [time frame: indicated within 6 h of birth; as per national guidelines]
18. Abnormal neurological examination at discharge clinical assessment recording abnormal findings — tone, reflexes, gag [time frame: up to first hospital discharge]
19. FBS-related injury/complication to baby (as reported on neonatal examination) — traumatic injury or abnormal bleeding [time frame: after birth]
20. Major obstetric haemorrhage >1000mL — postpartum haemorrhage [time frame: up to 24 h after birth]
21. Obstetric Anal Sphincter Injury (OASI — all degrees) injury either spontaneous or with episiotomy [time frame: at birth]
22. Referral to perinatal mental health services — psychological symptoms warranting referral [time frame: from birth up to 6 weeks after birth]
23. Maternal acceptability of procedure (defined by questionnaire) [time frame: from birth up to 7 days after birth]
24. Number of second-line tests (dFSS or FBS) each event (rather than samples taken) [time frame: during labour up until birth]
25. Number of inconclusive/uninterpretable dFSS procedures — no clear acceleration or variability borderline — [time frame: in labour up until birth]
26. Number of failed FBS procedures no sample or reliable result achieved [time frame: during labour up until birth]

### Participant timeline {13}

**Fig. 1:**
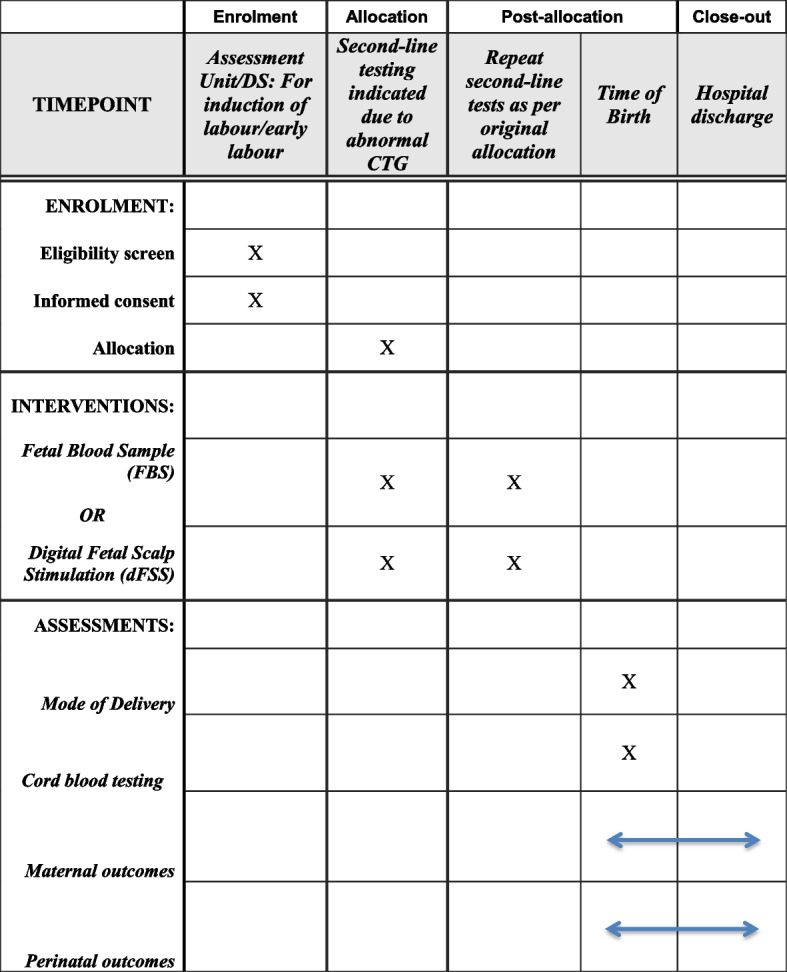
FIRSST trial schedule of enrolment, interventions and assessments

The participant timeline is presented in Fig. 1.

### Sample size {14}

Annual CS rates are between 25 and 35% in each centre although approximately 10% of these are elective. The caesarean section rate for nulliparous women in labour varies between 20 and 30% in each centre reflecting varying case mix and induction of labour policies. The eligible trial population includes only women who require continuous CTG monitoring (therefore excluding low-risk women) and only the subset of monitored women who have an abnormal CTG requiring a second-line test of fetal well-being. The CS rate is higher in this group of women as reflected in the feasibility and pilot study [14].

With 1250 women in each arm, the study will have 90% power to detect a difference of 5–6%, at a two-sided alpha significance level of 5%, assuming a CS rate of at least 20 % in the dFSS group. Allowing for a dropout rate or potential protocol violations in the initial recruitment phase for each centre, the total sample size required will be inflated by 10%. The pilot study suggested a very large difference in the incidence of CS between dFSS (20%) and FBS (52%). This strongly favours dFSS; however, the true effect in a large multi-centre setting may be smaller, and it is still possible that FBS may perform better than dFSS.

The Dublin Maternity unit and the Cork unit have approximately 7500 births a year with more than 80% of women receiving CTG in labour. Coombe Hospital statistics report that on average 10% of women have an FBS in labour with 780–800 women having FBS procedures (one or more) per year. The Limerick and Galway Maternity units have approximately 4500 and 2500 births a year respectively. Recruitment targets have been calculated to take account of site differences. The research staff will be funded to work 39 routine weekday hours. We estimate that 750 women will be recruited and randomised over a 30-month period in the two larger centres and 500 in each of the two remaining centres (total 2500). We anticipate that an inflated number of women will be recruited and consented, given the high use of CTG, and at least 2500 recruited and randomised women with adherence to the study protocol will be available for analysis at trial end.

The following calculations are based on a two-sided test.

**Table:** 

CS rate — dFSS	CS rate — FBS	Power	Sample size per arm	Total sample size	**Total sample size plus 10% for missing data (sample size per arm)**
20%	26%	90%	1032	2064	2298 (1149)
25%	31%	90%	1175	2350	2614 (1307)
20%	27%	90%	769	1538	1710 (855)
25%	32%	90%	872	1744	1938 (969)
30%	37%	90%	954	1908	2120 (1060)

### Recruitment {15}

Potentially eligible women will receive information at a routine antenatal clinic visit, at birth preparation classes and on admission for induction or in early labour. Posters informing potential participants and healthcare staff about the study will be placed in the relevant clinical areas. We will monitor recruitment and provide feedback to individual centres with regular newsletters once the study has been established. Recruitment targets have been set for each centre and will be reviewed at the Trial Steering Committee and Data Monitoring meetings.

## Assignment of interventions: allocation

### Sequence generation {16a}

#### Allocation to trial groups

Allocation of eligible women who consent to participate in the trial will be generated using a fully automated password-protected computer-based system provided by the HRB Centre for Primary Care Research, Royal College of Surgeons in Ireland. The randomisation sequence will be created using random permuted blocks of varying size and stratified by centre, in a 1:1 ratio for FBS versus dFSS.

### Concealment mechanism {16b}

The allocation sequence is concealed by the use of a password-protected automated computer-based randomisation platform.

### Implementation {16c}

The allocation sequence is generated by the password-protected computer-based randomisation platform. The participants will be enrolled by a trained doctor or midwife. Once written consent has been given, confirmation that all criteria are met and that the woman is happy to continue in the study, the doctor/midwife will obtain the randomised allocation.

## Assignment of interventions: blinding

### Who will be blinded {17a}

Trial participants and healthcare providers cannot be blinded to the interventions. Outcome assessors will complete the recording of the primary outcome and secondary maternal and perinatal morbidity outcomes without knowledge of the allocation. Only then will data be recorded in relation to the second-line testing in labour for the procedural outcomes, including whether there was any deviation from the allocated second-line test. Data analysis will be conducted by the trial statistician blind to the allocated intervention.

### Procedure for unblinding if needed {17b}

The design is open label with only outcome assessors and data analysts being blinded, so unblinding will not occur.

## Data collection and management

### Plans for assessment and collection of outcomes {18a}

Data will be collected on a standardised case report form (CRF) by a trained research doctor or midwife. The researcher will be responsible for ensuring that the details of the delivery are recorded and documented according to the study protocol. The inpatient maternal and neonatal notes will be marked so that they can easily be recovered following discharge from the hospital if required (unless electronic records are only in use). After discharge, the CRF will be collected by the local co-ordinator and the completeness of the data checked. Any errors or omissions will be followed up at this time. The randomisation allocation will be stored separately from the electronic database and will only be forwarded to the study statistician upon request.

### Plans to promote participant retention and complete follow-up {18b}

Participants will be recruited and consented as close as possible to the time of randomisation which will promote participant retention. However, labouring women are a particularly vulnerable group and written informed consent will be taken in early labour or prior to commencing induction of labour. The important step will be to ensure that eligible women are randomised when second-line testing is indicated. This will be reinforced with ongoing health professional education about the study and feedback on recruitment statistics. Follow-up will be complete at the time of hospital discharge and loss to follow-up should be minimal.

### Data management {19}

Data from paper CRFs will be entered into an electronic database (password protected) by the research doctor/midwife at each of the participating sites. Electronic CRFs will have participant identifying details removed and be labelled with coded identifiers. Initially, 10% of paper CRFs will be crossed checked against electronic CRFs for accuracy by the trial co-ordinator, with double data entry indicated if any important errors are identified. Data discrepancies will be discussed with the research personnel and amended by the site accordingly.

### Confidentiality {27}

Hard copy CRFs will be stored in a locked filing cabinet at each participating site and only the research midwife/fellow at each of the participating sites will have access. Data from paper CRFs will be entered into an electronic database (password protected) by the research doctor/midwife at each of the participating sites. Electronic CRFs will have participant identifying details removed and be labelled with coded identifiers before transfer to the main co-ordinating site on the master database.

### Plans for collection, laboratory evaluation and storage of biological specimens for genetic or molecular analysis in this trial/future use {33}

This trial does not involve collecting biological specimens for storage.

## Statistical methods

### Statistical methods for primary and secondary outcomes {20a}

**Fig. 2:**
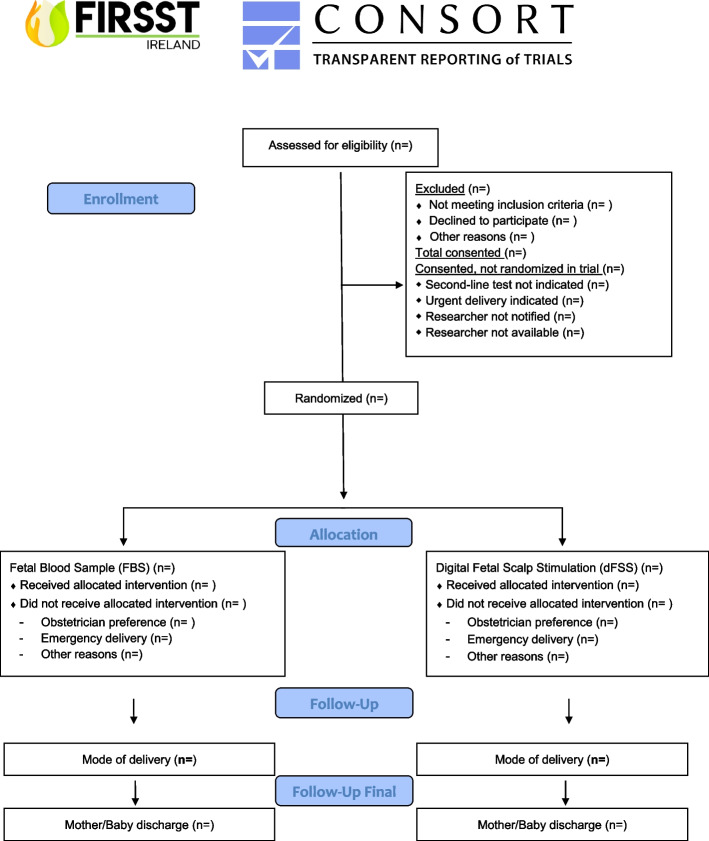
CONSORT flow diagram of the FIRSST trial

Data analysis and reporting will proceed according to CONSORT guidelines for randomised controlled trials and will be conducted blinded to group allocation by the trial statistician and researcher (Fig. 2). The first stage of analysis will be to use descriptive statistics to describe recruited individuals in relation to those eligible and to investigate comparability of the trial arms at baseline. The primary analysis will involve an intention-to-treat (ITT) comparison between the two groups for the primary outcome adjusted for stratification factors — this will be the study centre. The ITT population will include all randomised patients according to the treatment they were randomised to receive. Secondary outcomes will be analysed in a similar way. The primary outcome measure is caesarean section (CS). In all cases, the CS will be performed in labour and in the context of an abnormal CTG. We will compare the proportion of women delivered by CS using ITT, as well as per-protocol analysis. The per-protocol population will consist of all randomised patients who have adhered to the allocated treatment and were not lost to follow-up and who have no major protocol deviations.

Given the nature of the trial, there is a possibility of deviations from allocation. For example, if the dFSS is inconclusive, uninterpretable or abnormal, the obstetrician may proceed to FBS. The number (and percentage) of patients with deviations will be summarised by treatment group with details of the type of deviation provided. The patients that are included in the ITT analysis data set will be used as the denominator to calculate the percentages. Additionally, further analysis of the primary and secondary outcomes will be conducted where participants will be analysed according to the treatments they actually received (dFSS alone, FBS alone, dFSS and FBS) and the impact assessed and reported.

All analyses will use appropriate (that is, logistic or linear) regression models, with results presented as point estimates, 95% confidence intervals (CI) and *p*-values. The difference in the primary endpoint between the two groups will be expressed as an odds ratio (OR) and 95% CI. Further secondary analyses will involve planned subgroup analyses.

The economic evaluation for this study will be informed by a decision analytical model which will be designed and constructed for the study to reflect the maternal and fetal pathways and health states. Employing a decision analytical model allows for the extrapolation of existing data and the opportunity to systematically synthesise evidence from various sources. Primary data on maternal health outcomes will be available from the study and will include CS rates, AVB rates as well as duration of admission and postnatal complications. Perinatal outcomes, such as fetal acidosis, neonatal intensive care unit admission and encephalopathy, will be collected during the study and their appropriate conversion to health-related utilities will be informed by a review of the literature. Resource use will be collected using a costing questionnaire and the Hospital In-Patient Enquiry (HIPE). The costs and effects of the intervention (dFSS) and comparator (FBS) will be compared to estimate an incremental cost-effectiveness ratio (ICER) in a cost-utility analysis. To address each parameter and structural uncertainties, a probabilistic sensitivity analysis (PSA) will be performed.

### Interim analyses {21b}

An independent safety and data monitoring committee (DMC) will meet yearly to examine recruitment figures, baseline data, retention and adverse events. No formal interim analyses for either safety or effectiveness are planned and therefore there are no formal stopping rules. The two second-line tests under evaluation are used routinely in Irish maternity units. However, all serious adverse events (SAEs) will be reported to the DMC who will report back to the Trial Steering Committee. Additionally, health professionals caring for the patient are free to manage the patient according to best clinical practice as dictated by the lead clinician in keeping with the clinical circumstances.

### Methods for additional analyses (e.g. subgroup analyses) {20b}

Subgroup analyses will use multivariable regression models with appropriate interaction terms to ascertain any differential effects in relation to onset of labour (spontaneous or induced), use of oxytocin (infusion or no infusion) and presence of meconium (present or absent).

### Methods in analysis to handle protocol non-adherence and any statistical methods to handle missing data {20c}

The primary and most of the secondary outcomes are mandatory fields in the electronic patient records and we do not anticipate missing data for these items. While paired cord blood samples should be taken and tested following birth in all cases where CTG abnormalities requiring second-line testing are detected, we anticipate that there may be some missing data where samples are insufficient or not taken. The analyses for this outcome will be restricted to the sub-sample with reliable data.

### Plans to give access to the full protocol, participant-level data and statistical code {31c}

The full protocol, participant-level data and statistical code will be available from the corresponding author on reasonable request.

## Oversight and monitoring

### Composition of the coordinating centre and trial steering committee {5d}

The trial management group co-ordinating the study is multi-disciplinary including the disciplines of obstetrics, midwifery, neonatology, statistics, trials methodology and public and patient involvement (PPI). The trial will be overseen by a Trial Steering Committee (TSC) and Data Monitoring Committee (DMC) who will meet at least annually. The clinical research facility (CRF) at the co-ordinating institution will provide formal monitoring and audit of trial conduct. The TSC will provide independent supervision for the trial and advice to the chief investigator and principal investigators and to the sponsor on all aspects of the trial. The regulatory processes will ensure protection for participants by ensuring the trial is conducted according to the guidelines for Good Clinical Practice.

### Composition of the data monitoring committee, its role and reporting structure {21a}

An independent data monitoring committee (DMC) will include clinician and statistician members. They will meet at least annually. The objective of the DMC is to provide an impartial and objective assessment of the trial data and advise the Trial Steering Committee (TSC) on the need for continuing or stopping the trial. The recommendation may be to do one of the following:

- Continue the trial as planned
- Continue but amend the protocol prior to moving forward with the trial
- Stop the trial

### Adverse event reporting and harms {22}

All intrapartum adverse events will be reported in the usual way as critical incidents and reviewed in accordance with the local risk management procedures. From the trial perspective, serious adverse events (SAEs) will be recorded and reported to the regulatory authorities. SAEs include perinatal death, neonatal encephalopathy and neonatal encephalopathy requiring therapeutic hypothermia. In the event of a SAE occurring, a form will be completed by the local researcher and faxed to the trial co-ordinating centre at the Coombe Women’s Hospital within 72 h. The chief investigator will inform the Chair of the Data Monitoring Committee (DMC) and the Chair of each REC will also be informed by the DMC Chair if considered appropriate. The DMC procedures for dealing with SAEs and suspected unexpected serious adverse reactions (SUSARs) are outlined in the DMC Charter.

### Frequency and plans for auditing trial conduct {23}

A data monitoring plan is in place with a total of 16 site visits across the four participating sites auditing the procedures and conduct of the trial. This will be independent of the investigators and sponsor.

### Plans for communicating important protocol amendments to relevant parties (e.g. trial participants, ethical committees) {25}

Important protocol modifications (e.g. changes to eligibility criteria, outcomes, analyses) will be discussed with the TSC and communicated to all relevant parties as appropriate (e.g. investigators, RECs, trial participants, trial registries, journals, regulators).

## Dissemination plans {31a}

We aim to raise awareness of this clinical question and the proposed research approach at local, national and international meetings. A final report will be prepared for the funding body and papers will be prepared for open access peer-review publication. We plan to include the trial results in an updated edition of the Cochrane review that we are leading. We will liaise with national and international guideline developers (HSE/RCOG/NICE/WHO) to ensure that the trial findings are incorporated into evidence-based practice guidelines and reach the target audience as early as possible.

## Discussion

It is both important and timely that we evaluate the optimal approach to second-line testing of fetal wellbeing in the context of CTG abnormalities in labour. Rates of caesarean section in labour have risen over the last two decades and continue to rise. Safe birth and wellbeing of the mother and baby are the priorities for maternity services. Pregnant women, midwives and obstetricians need high-quality evidence on which to base management decisions. Despite its widespread use, the optimal approach to second-line testing of fetal wellbeing in labour is a poorly evaluated component of maternity care. The overall aim of this project is to reduce unnecessary interventions, particularly caesarean section, and the consequences of operative birth for the current and future pregnancies. In addition, we will address the economic implications of the two approaches being evaluated.

## Trial status

Protocol version 9.0. Date: 03.08.2022

Enrolment commenced: 10.05.2022

Recruitment completed approximately: 2025

## Data Availability

All data and materials will be available from the corresponding author on reasonable request.

## Abbreviations

CTG: Cardiotocography
FBS: Fetal blood sampling
dFSS: Digital fetal scalp stimulation
CS: Caesarean section
AVB: Assisted vaginal birth
SVB: Spontaneous vaginal birth
DDI: Decision delivery interval
NNU: Neonatal unit
PPH: Primary postpartum haemorrhage
BMI: Body mass index
RCT: Randomised controlled trial
TSC: Trial Steering Committee
DMC: Data Monitoring Committee
SAE: Serious adverse event
SUSARs: Suspected unexpected serious adverse reactions
